# Chemodynamic Therapy of Glioblastoma Multiforme and Perspectives

**DOI:** 10.3390/pharmaceutics16070942

**Published:** 2024-07-15

**Authors:** Zia Ullah, Yasir Abbas, Jingsi Gu, Sai Ko Soe, Shubham Roy, Tingting Peng, Bing Guo

**Affiliations:** 1 School of Science, Shenzhen Key Laboratory of Flexible Printed Electronics Technology, Shenzhen Key Laboratory of Advanced Functional Carbon Materials Research and Comprehensive Application, Harbin Institute of Technology, Shenzhen 518055, China; ziadrishak@gmail.com (Z.U.); yasirabbas56778@gmail.com (Y.A.); kaunggyi711@gmail.com (S.K.S.); shubham.royju@gmail.com (S.R.); 2 Education Center and Experiments and Innovations, Harbin Institute of Technology, Shenzhen 518055, China; gujingsi@hit.edu.cn; 3 State Key Laboratory of Bioactive Molecules and Druggability Assessment, College of Pharmacy, International Cooperative Laboratory of Traditional Chinese Medicine Modernization and Innovative Drug Development of Ministry of Education (MOE) of China, Jinan University, Guangzhou 511436, China

**Keywords:** chemodynamic therapy, Fenton reaction, Fenton-based nanomaterials, glioblastoma multiforme, reactive oxygen species

## Abstract

Glioblastoma multiforme (GBM), a potential public health issue, is a huge challenge for the advanced scientific realm to solve. Chemodynamic therapy (CDT) based on the Fenton reaction emerged as a state-of-the-art therapeutic modality to treat GBM. However, crossing the blood–brain barrier (BBB) to reach the GBM is another endless marathon. In this review, the physiology of the BBB has been elaborated to understand the mechanism of crossing these potential barriers to treat GBM. Moreover, the designing of Fenton-based nanomaterials has been discussed for the production of reactive oxygen species in the tumor area to eradicate the cancer cells. For effective tumor targeting, biological nanomaterials that can cross the BBB via neurovascular transport channels have also been explored. To overcome the neurotoxicity caused by inorganic nanomaterials, the use of smart nanoagents having both enhanced biocompatibility and effective tumor targeting ability to enhance the efficiency of CDT are systematically summarized. Finally, the advancements in intelligent Fenton-based nanosystems for a multimodal therapeutic approach in addition to CDT are demonstrated. Hopefully, this systematic review will provide a better understanding of Fenton-based CDT and insight into GBM treatment.

## 1. Introduction

Glioblastoma multiforme (GBM), a potential public health issue that is hiking the mortality rate worldwide, is a malignant brain cancer. Currently, available treatment fidelities for GBM include surgery, radiation therapy, chemotherapy, immunotherapy, photodynamic therapy, photothermal therapy, and gene therapy [1]. The conventional treatment modalities are less effective and have long-lasting adverse side effects even for the initial stages [2]. In the current era of advanced technology, it is a potential challenge to devise advanced and effective treatments for GBM with reduced aftereffects and increased efficacy for improved therapeutic outcomes. Chemodynamic therapy (CDT), the cutting-edge advanced cancer treatment modality, has attracted a lot of attention as a novel treatment fidelity for GBM because of its significant selectivity, decreased overall toxicities and complications, and no need for any activation stimulus required during treatment. Zhang et al. studied the pH dependent release of Fe^2+^ in amorphous iron nanoparticles (AFeNPs) and iron nanocrystals (FeNCs). The AFeNPs exhibited 100% ionization of Fe^2+^ at pH 5.4 as compared to pH 7.4, demonstrating high selectivity of CDT [3].

**Scheme 1:**
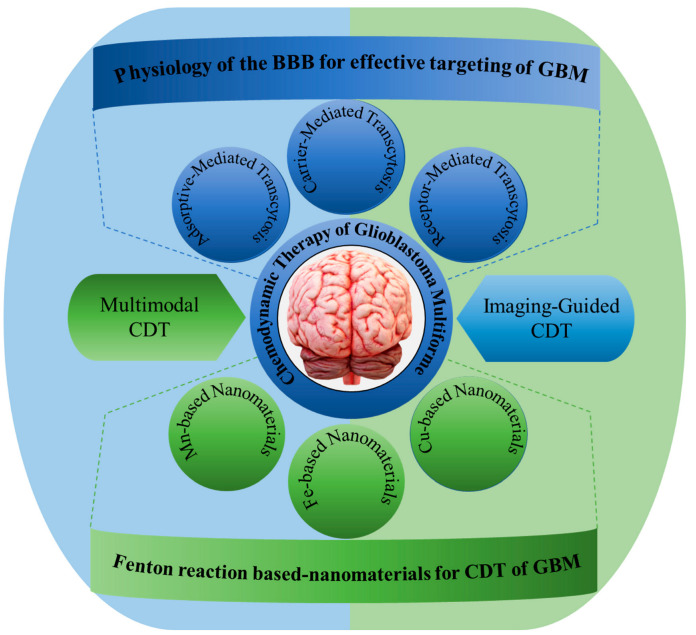
Schematic illustration of Fenton-based CDT of GBM by applying biocompatible smart nanomaterials to cross the BBB through vascular transport channels for effective targeted therapy.

CDT includes the introduction of nanocatalytic medications of transition metals to liberate metal ions into tumor cells to induce Fenton and Fenton-like reactions (Scheme 1). The breakdown of hydrogen peroxide, which is mediated by metal ions, to produce hydroxyl radicals (^•^OH) is known as the Fenton reaction [4]. Reactive oxygen species (ROS) are produced and accumulated through the Fenton reaction, which is the therapeutic mechanism of CDT that causes cancer cell death [5]. However, the ROS production in CDT is not only limited to Fenton reactions. Researchers have devoted their major efforts to understand the mechanism of ROS production in biological systems. For the production of singlet oxygen (^1^O_2_), the reactions of peroxides (H_2_O_2_ and lipid hydroperoxides) have been proven [6]. Moreover, the ^1^O_2_ can be produced by the reaction of biological hydroperoxides in the presences of trace amount of metal ions or enzymes known as the Russel mechanism. Besides all these discoveries, there is still a need to understand the complex mechanism of ROS production in CDT [7].

Compared to conventional treatments, CDT can produce tissue depth-unlimited, spatiotemporal, and controllable ROS and O_2_ as a result of the elevated concentration of H_2_O_2_ within the tumor microenvironment (TME) by using transition metal nanoparticles [8,9]. To better understand the therapeutics of GBM, it is mandatory to objectively understand the physiology of blood–brain barriers (BBB) [9]. The BBB serves as a highly precise and flexible contact between blood vessels and the central nervous system and acts as the largest barrier to the blood circulation system to carry drugs to the central nervous system [10]. To cope with this potential challenge in CDT of GBM, scientists have engineered nanoparticle-based drug transport systems by using natural influx transporters present on the BBB that can effectively penetrate the BBB [11]. These strategies include cell-mediated transport, BBB disruption-enhanced transport, adsorptive-mediated transcytosis (AMT) [12,13], carrier-mediated transcytosis (CMT) [14], and receptor-mediated transcytosis (RMT) (Scheme 1) [13].

This review will briefly discuss the physiology of the potential BBB and demonstrate the mechanism of CDT for the treatment of GBM. Recently, multimodal techniques that combine CDT with other therapies have been developed, such as CDT-Immunotherapy, CDT-Chemotherapy, CDT-Sonodynamic, and multimodal therapies are also considered in this review. Subsequently, difficulties and the future perspectives of CDT are summarized for further possible advancements in the treatment of GBM.

## 2. Physiology of Blood–Brain Barriers (BBB): Potential Barriers to the Brain

The potential barriers to the brain, BBB have the following components: the basement membrane, pericytes, perivascular astrocyte end-foot processes, tight junctions, adherents intersections, an ongoing layer of non-fenestrated capillary endothelial cells surrounded by the glycocalyx, a network of intercellular tight junctions, and adherents intersections [15]. Three main barriers in BBB are positioned in a sequence having the extravascular section, the epithelium, and the glycocalyx go from the bloodstream to the brain in that order. Understanding the composition and operation of these barriers is crucial for developing new medications that can effectively target invasive tumor cells as well as for comprehending the use of optic indicators in brain tumor therapeutics associated with a healthy brain [16,17]. By blocking the entry of medications, inflammatory cells, blood-borne proteins, and other solutes, the BBB secures the sensitive surroundings of the brain. Consequently, this also inhibits the transport of fluorescent markers into the brain, decreasing the efficacy of optical imaging methods [18].

**Figure 1:**
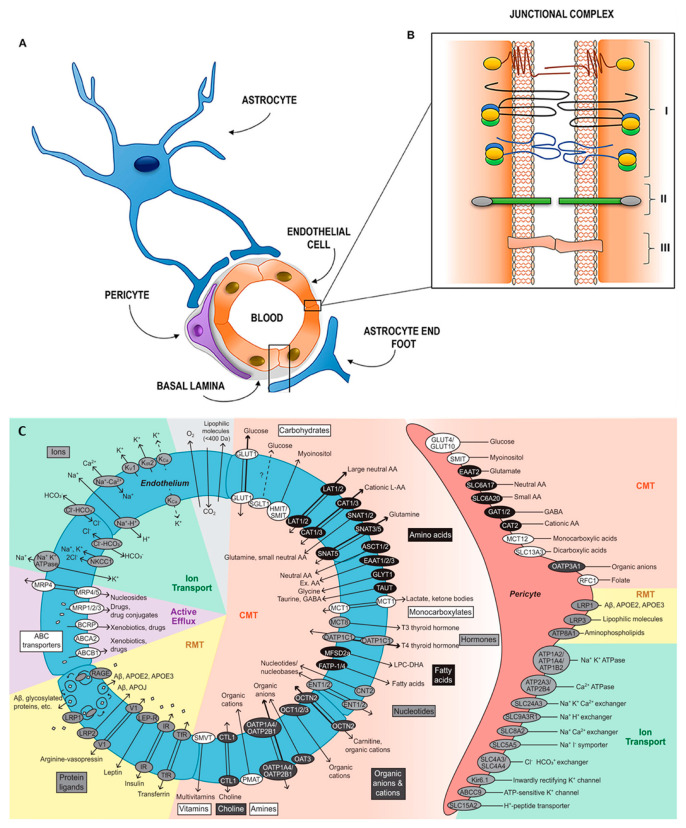
Schematic representation of BBB and structure of transport pathways across the BBB. (**A**) Diagrammatic representation of potential BBB of the neurovascular system. (**B**) The complicated junctional complex of the BBB: (I) tight junctions; (II) adherents junctions; (III) GAP junctions [23]. Reused under Creative Commons Attribution License. (**C**) The structure of major BBB transport pathways present in the neurovascular system. The transport pathways include carrier-mediated transport, receptor-mediated transport, ion transport, and active flux [24]. Reused under Creative Commons Attribution License.

By making use of the widely recognized endogenous BBB trafficking channels, a variety of nanoplatforms, including polymeric nanoparticles, micelles, liposomes, protein nanocages, and inorganic nanoparticles, are successful in regional brain tumor administration [19]. Here is a comprehensive introduction of benefits of nanomedicine over conventional therapy include better biocompatibility, multiple drug loading ability, cell or tissue targeting ability, controlled drug release ability, and even the capacity to traverse the BBB [20]. Enhancing the size of the nanoparticle, modulating the shape, varying the ligand density, increasing the lipophilicity, and surface chemical modification can also effectively intensify the amounts of nanoparticles accumulated in the brain and improve the therapeutic effect. The brain-targeted systems that have been investigated the most are nanoparticles, polymeric micelles, and liposomes [17]. These systems can regulate the release of encapsulated pharmaceuticals, shield them from biological and chemical deterioration in the bloodstream, allow surface modification with targeted ligands, and facilitate PEGylation or steric stability. Because liposomes resemble the structure of the cell membrane and are composed of naturally occurring biological lipids, they are regarded as lowly toxic and biologically friendly [21]. Nanocarriers loaded with potent chemotherapeutic agents can elegantly traverse the BBB through carrier-mediated, receptor-mediated, adsorption-mediated, and cell-mediated transport mechanisms, ensuring precise delivery of therapeutic drugs to the desired site for optimal treatment outcomes (Figure 1) [22]. These features render nanomedicine a compelling therapeutic modality for brain tumors.

For further enhancement of the therapeutic efficacy of CDT for the treatment of GBM, the nanomaterials having the ability to cross the BBB with maximum therapeutic efficiency need to be formulated by the researchers. There is also a need to engineer smart nanomaterials that have targeting ability and can only be activated in the tumor area with maximum biocompatibility.

## 3. Mechanism of ROS Generation

Prominent ROS comprise superoxide anion (O^2−^), hydroxyl radical (^•^OH), and singlet oxygen (^1^O_2_) and they act as regulatory and signaling molecules in the cell [25]. A moderate level of ROS is necessary to regulate some important physiological processes such as enzyme activation, expression of genes, and intracellular trafficking. However, increased levels of ROS can cause oxidative damage to the cell by inactivation of the protein, peroxidation of phospholipids, and DNA damage [26]. It is well known that TME exhibits hypoxia condition, intracellular overexpression of H_2_O_2_ and glutathione (GSH), lower pH, aberrant blood vessels, and enhanced consumption of cell nutrients than the healthy body parts [27]. To greatly increase intracellular ROS in the cancer cells to enhance the oxidative stress resulting in apoptosis and necrosis, CDT typically uses Fenton and Fenton-like reactions, metallic catalysis, or peroxidase-like catalysis [28]. Here, Fe, Cu, Mn, Ag, Ru, W, Ce, Co, V, and Pd are a few of the metal ions that are present in transitional metal-based nanocatalytic medicines having the ability to start Fenton and Fenton-like processes that change H_2_O_2_ into ROS, which is more toxic in an acidic TME, ultimately killing tumor cells [29].

The lower pH (6.5–6.9) of TME and overexpressed H_2_O_2_ (approx. 100 μM) provides a suitable environment for the Fenton reaction to occur [30]. Because of short-lived ^•^OH (10^−9^ s), it is primarily necessary to engineer Fe-based nanomaterials for noninvasive intracellular Fenton reactions having targeted therapeutic ability. Scientists have designed several nanomaterials and nanozymes by using different transition metal ions (Mo^4+^, V^2+^, Cu^+^, Ag^+^, and Mn^2+^) [31,32,33] to reduce the requirement for lower pH. For instance, the Cu-based Fenton reaction can occur 160 times faster than that of the Fe-based reaction to produce ^•^OH in the TME [34]. Moreover, the Cu^2+^ produced during the Fenton reaction can be reduced back to Cu^+^ by endogenous overexpressed GSH. The GSH maintains the intracellular redox hemostasis and thus decreases its concentration promoting the Fenton reaction. The Fenton reaction by such kind of transition metals has several benefits, like the higher occurrence of structurally different oxide compounds and optimum performance in lower charge conditions [29]. However, low pH and higher concentration of catalyst are required for Fe-based nanomaterials and they have optimal performance at low activation energy and lower H_2_O_2_ concentration as compared to other species. Before engineering the nanomaterial for the Fenton reaction, it is mandatory to consider the feasibility of an active redox cycle in specific pH, loading of catalyst, and oxidation product stability. The challenge of designing a full package of chemodynamic material and the complexity of TME are the potential barriers to CDT [35,36]. For example, (1) tumors consisting of 50–100 μM H_2_O_2_ lack adequate endogenous levels, resulting in suboptimal therapeutic efficiency of CDT. (2) The excessive generation of reducing agents in TME, like GSH and H_2_S, may absorb and neutralize the ^•^OH, leading to inadequate therapeutic efficacy. (3) Solid tumors need to be altered to produce optimum reaction circumstances due to their mild acidity which makes them unsuitable for Fenton reaction. Therefore, the structure of the nanomaterial for the Fenton reaction and TME modulation should be in favor of CDT. To date, numerous nanozymes and approaches have been designed to address these challenges [37].

## 4. Design of the Nanomaterial for CDT

After a comprehensive understanding of the physiology of the BBB and the mechanism of ROS generation, the nanomaterials can efficiently be engineered for efficient Fenton reaction to enhance the therapeutic efficiency of CDT for GBM. The two main approaches that can be adopted for enhanced CDT are enhancement of the electron transfer for efficient Fenton reaction and tumor targeting ability of the nanomaterial which enable them to accumulate in the tumor area for effective ^•^OH generation [38].

The short life span and limited diffusion range of ^•^OH within the TME is one of the pronounced issues of CDT. To increase the cell death ratio, the higher concentration of ^•^OH can be directed toward vulnerable biomolecules in the cell by adopting a tumor-targeting approach to deliver the therapeutic nanomaterials directly into the cells [39]. Recently, to increase the spatiotemporal CDT efficiency, Qiao et al. engineered traceable multistage targeting nanomaterials. The mitochondria and tumor-targeting ability were introduced in the nanomaterial by incorporating triphenylphosphine (TPP) and biotin. For targeted CDT, the nanomaterial effectively delivered α-tocopheryl succinate and lonidamine showing in vivo tumor ablation. Moreover, the scientists utilized cell membrane-coated nanomaterials for increasing biocompatibility and tumor targeting [40].

**Figure 2:**
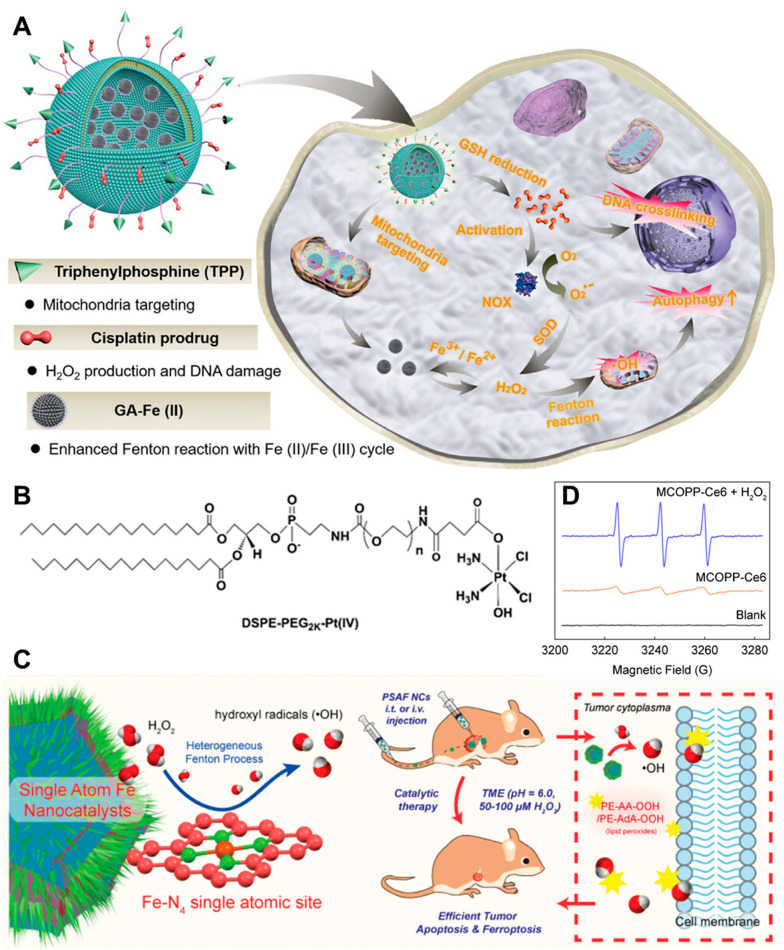
(**A**) Schematic demonstration of the in vivo multimodal sequential CDT based on mitochondria targeting nanomaterials. The level of endogenous H_2_O_2_ was elevated through the activation of NOX-associated cascade reaction via bioactive cisplatin. The Fe-based nanocomposite subsequently catalyzed H_2_O_2_ into toxic ^•^OH to induce autophagy and inhibited tumor progression. (**B**) Chemical structure of bioactive cisplatin (DEPE-PEG_2k_-Pt (IV)) [43]. Reproduced with permission from [43] Copyrights 2021, WILEY. (**C**) Schematic representation of cancer therapy catalyzed by Fe-based nanomaterials [44]. Reproduced with permission from [44] Copyrights 2020, WILEY. (**D**) ESR spectrum of ^1^O_2_ entrapped by TEMP [45]. Reproduced with permission from [45] Copyrights 2020, AMERICAN CHEMICAL SOCIETY.

The electron density and reaction activation energy can be modulated by the functionalization of the nanomaterial to change the chemical potential of the active electrons. For the Fenton reaction, the Fe^3+^/Fe^2+^ redox cycling byH_2_O_2_ is essential. The rate-determining step for a continuous supply of Fe^2+^ for H_2_O_2_ decomposition to produce ^•^OH and decrease the energy of activation for Fe^3+^ ion can be facilitated by incorporating electron-rich nanomaterials in the Fe-based CDT [41]. The electron density of Fe can be modulated by transferring charge from the non-reaction center atom toward the reaction center (Fe) atom. Apart from this, scientists have engineered Z-scheme heterojunctions (Figure 2). In this scheme, the electrons in the conduction band of the first junction can travel to the valance band of the other junction upon external stimuli to create electron supplementation and favor the ROS generation. A thermally oxidized pyrite nanosheets functionalized by PEG-NH_2_ with a Z-scheme heterojunction assembly has been synthesized by Ji et al. [42].

Moreover, the CDT efficiency of the Fenton reaction can be enhanced by employing single transition metal atom-based nanomaterials. The heterogenous catalysis is considered as a surface phenomenon and increasing the surface-active sites on a catalyst will increase the catalytic activity. In this context, single transition metal atom-based nanomaterials can demonstrate the maximum catalytic activity by using surface active sites, exhibiting increased ROS production [46].

## 5. Application of Nanomaterials in CDT of GBM

Research in designing effective nanomaterials for Fenton reactions has gained a lot of attention because of the widespread use of CDT in cancer treatment. Catalysts play a prime role in Fenton reactions because the selection of the right nanomaterials (Fe-based, Cu-based, and other metal-based nanomaterials) is crucial. In this section, the application of Fe-based, Cu-based, and Mn-based nanomaterials for CDT of the GBM is discussed. Table 1 depicts the recent advances in nanomedicines applied for CDT of GBM.

### 5.1. Fe-Based Nanomaterials for CDT of GBM

The Fenton reaction has been the basis for the design of numerous nanomaterials with catalytically active and biocompatible ions in recent years. Fe-based nanomaterials have demonstrated excellent biocompatibility and have been extensively utilized in the medical realm [57,58]. Numerous Fe-based nanomaterials, including Fe_3_O_4_ [59], FeS_2_ [59], Fe_2_O_3_ [60], Fe metal–organic frameworks (Fe-MOFs) [61], and Fe-doped nanoagents [62], have been thoroughly investigated to increase the effectiveness of CDT. Fe^2+^ or Fe^3+^ is frequently the catalyst of a Fenton reaction, which breaks down H_2_O_2_ to produce the radical ^•^OH and destroys proteins, DNA, and lipids. For example, Li et al. designed superparamagnetic iron oxide nanoclusters to effectively catalyze the Fenton reaction. The human serum albumin coating and conjugated RGD peptide ligands provided stability, biocompatibility, and efficient glioma targeting. A large number of cytotoxic ROS liberated from photothermally assisted Fenton reaction conjugated with chemotherapy resulted in glioma apoptosis upon NIR light illumination. The mentioned combinatory therapy was more effective in promoting glioma ablation than any single treatment. Considering the glioma growth inhibition, specific targeting ability, and biosafety, the synergistic chemo/chemodynamic treatment can be promising for use in the clinic [63].

**Figure 3:**
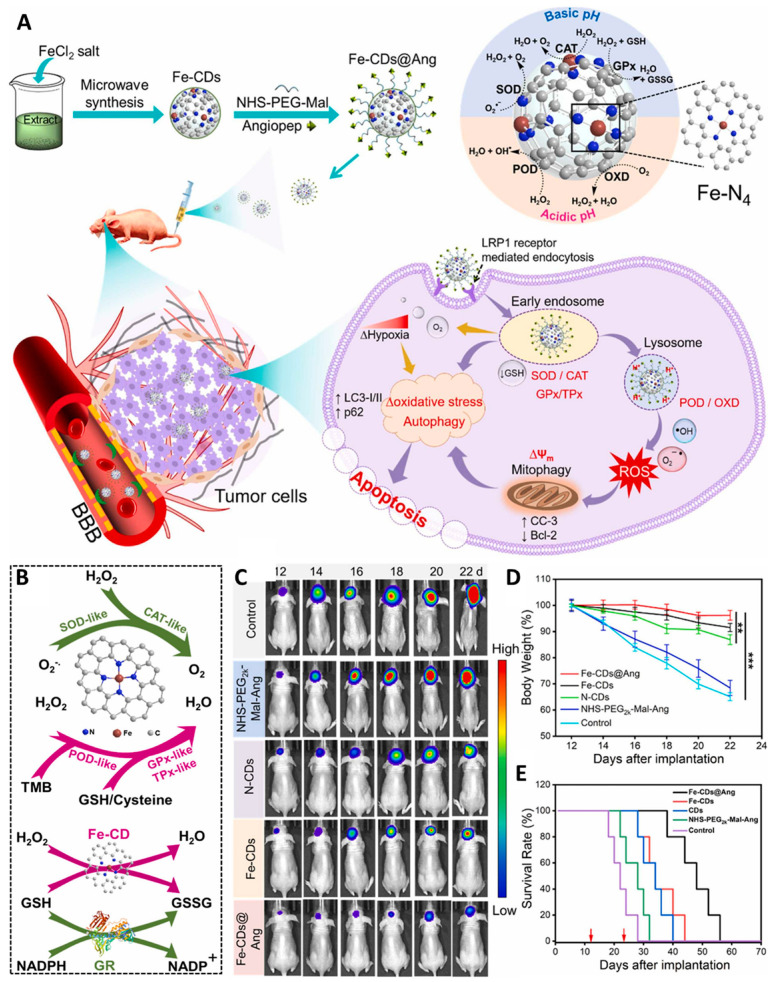
(**A**) Diagrammatic representation of Fe-based nanozyme preparation and enzyme-based cascade initiation by angiopep-2 and Fe-based nanozyme modification for ROS generation to induce the lysosome-based autophagy for the therapy of GBM. (**B**) Diagrammatic representation of enzymatic mimicking via Fe-based nanozyme. The removal process of H_2_O_2_ by using Fe-based nanozyme with increased properties like GPx under the glutathione reductase coupled process. (**C**) Fluorescence images of nude mice bearing orthotopic tumor following treatment different samples. (**D**) Demonstration of the changes in the body weight of the tumor-bearing mice after the treatment with Fe-based nanozymes. (n  =  6, one-way ANOVA and Tukey multiple comparisons tests, ** *p* < 0.05, *** *p* < 0.001) (**E**) Demonstration of the survival rates of the tumor-bearing mice after the treatment with Fe-based nanozymes [66]. Reproduced with permission from [66] Copyrights 2022, ELSEVIER.

A nanozyme of ultra-small Fe-doped carbon dots (Fe-CDs) was created at the atomic level by controlling the single-atom Fe core with precise coordination of nitrogen and carbon. Angiopep-2 was used to activate Fe-CDs to further facilitate accurate transport across the BBB. Synthetic ligands for the low-density lipoprotein receptor-related protein 1 (LRP-1), such as Angiopep-2, are generated from the Kunitz domain and have been demonstrated to gather GBM in the brain parenchyma and have a better transcytosis capacity as BBB shuttle peptides [64]. The Fe-CDs@Ang specifically aim at the LRP-1, which is highly expressed in brain capillary endothelial and neoplastic cells. As a result, a notable reduction in tumor growth was achieved by effectively targeting brain tumors with nanozymes utilizing LRPs-mediated transcytosis/endocytosis to cross the BBB. The Fe-CDs@Ang nanozyme targets ROS in TME, leading to tumor regression in GBM models (Figure 3). The Fe-CDs nanozyme mimics the intracellular ROS-mediated death pathway in drug-resistant GBM cells, exhibiting six kinds of enzyme-like activities. Studies have shown that Fe-CDs@Ang nanozymes effectively target tumors, penetrate the BBB, and induce autophagy-mediated cell death. Drug-free nanomedicine offers high efficacy and low toxicity in treating malignant GBM. When used in combination, multipurpose nanozyme Fe-CDs are recognized as a potent toolbox for the specific purpose of treating drug-resistant GBM [65].

The metal-based inorganic nanomaterials and metal–organic framework are prone to elicit toxic effects in the brain when used as CDT nanocatalysts. Moreover, most of the conventional Fenton nanomaterials are not biodegradable and may remain in the body for a significant period and can cause serious complications to the brain. Thus, it is necessary to engineer highly biocompatible nanomaterials to reduce neurotoxicity [67]. To overcome this neurotoxicity caused by the inorganic/organic nanomaterials, Zheng et al. designed a novel CDT nanomaterial. They utilized natural metalloproteins containing metal ions (Fe^2+^, Cu^2+^, Mn^2+^, and Co^2+^) as cofactors and can act as Fenton catalysts. The hemoglobin (Hb) containing Fe was selected as a Fenton catalyst and glucose oxidase (GOx), having the ability to generate H_2_O_2_, acted as a complementary part to complete the Fenton reaction. The major advantages of using Hb and GOx as Fenton catalysts for CDT are excellent biocompatibility and biodegradability [68]. The assembling and crosslinking methodology was employed to formulate the Hb and GOx protein superstructures. The superstructure acted as a self-delivery agent, eliminating the requirement for extra drug delivery agents. Furthermore, the protein superstructures were coated with red blood cell (RBC) membranes to reduce the immunogenicity, which greatly enhanced the blood circulation time, and helped to cross the BBB. They successfully demonstrated that RBC@Hb@GOx nanomaterials are promising nanomaterials for the treatment of GBM [69].

### 5.2. Cu-Based Nanomaterials for CDT of GBM

More significantly, TME with particular properties can be employed to support intelligent nanomaterials having maximum efficiency and minimal invasiveness [70]. It has been demonstrated that Cu-based nanomaterials can deplete GSH and catalyze the transformation of H_2_O_2_ into ROS (^•^OH) for CDT of GBM.

**Figure 4:**
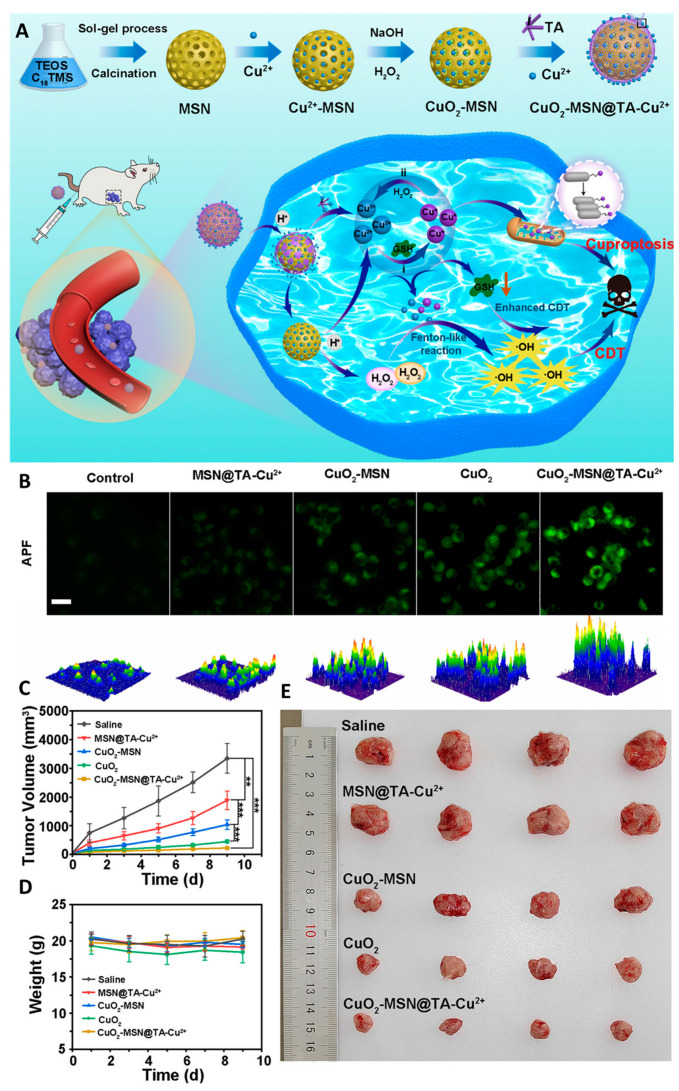
(**A**) Diagrammatic representation of employing Cu-based nanomaterials for cuproptosis and CDT. (**B**) The generation of ROS by the Cu-based nanomaterial at different concentrations. (**C**) After treatment by Cu-based nanomaterials, the tumor volume curve of the tumor-bearing mice. (Scale bar: 25 μm) (**D**) After treatment by Cu-based nanomaterials, the body weight of the tumor-bearing mice. (**E**) Demonstration of therapeutic efficiency of the Cu-based nanomaterials [71]. Reproduced with permission from [71] Copyrights 2024, AMERICAN CHEMICAL SOCIETY.

Tian et al. designed an intelligent Cu-based nanomaterial, synthesized by in situ formation of CuO_2_ in the mesoporous silica nanoparticles (MSN) and then coated with a tannic acid (TA)-Cu^2+^ complex for Cu-based CDT. The Cu-based nanomaterial (CuO_2_-MSN@TA-Cu^2+^) demonstrated a TME-triggered therapeutic approach. The nanomaterial has dual functionality, the outer TA-Cu^2+^ complex quickly disintegrated to liberate Cu^2+^ and release the inner CuO_2_ to produce H_2_O_2_ and Cu^2+^. The Cu-based nanomaterial not only converted endogenous and self-supplied H_2_O_2_ into toxic ^•^OH radical via Cu-based Fenton reaction for CDT but also underwent GSH-mediated Cu^+^ reduction to induce potential cellular curoptosis and enhanced CDT. The results indicated that CuO_2_-MSN@TA-Cu^2+^ produced a remarkable cytotoxicity against the cancer cells and greatly suppressed the tumor growth up to 93.24% in a mice model (Figure 4) [71].

**Figure 5:**
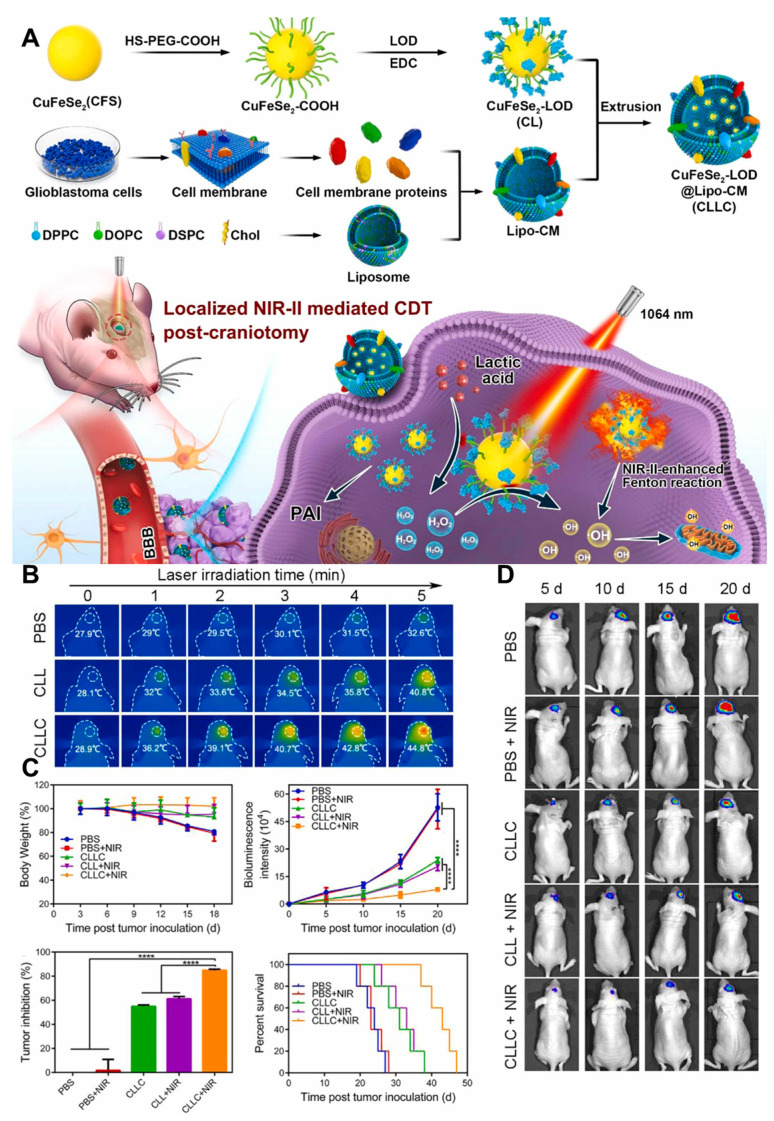
(**A**) Graphical representation of localized NIR-II laser-assisted CDT post-craniectomy. The laser excitation increased the temperature and further accelerated the Fenton reaction to speed up the production of ^•^OH. The extra ROS production induced mitochondrial polarization and causes cell apoptosis. (**B**) In vivo IR thermal images of the orthotopic GBM area pre- and post-excitation by 1064 nm laser. (**C**) Changes in the body weight of mice bearing orthotopic GBM, signal intensity of semiquantitative bioluminescence in the brain, inhibition analysis of mean tumor, and analysis of survival rates of mice bearing orthotopic GBM in each group. (**D**) Demonstration of bioluminescence images of mice bearing orthotopic U87MG tumor from each group [52]. Reproduced with permission from [52] Copyrights 2022, ELSEVIER.

Most of the therapeutic agents are ineffective for the treatment of GBM because these materials cannot cross the BBB effectively. Moreover, if the materials can cross the BBB, these cannot effectively target the GBM and cause serious toxicity in the brain. To address these drawbacks, Pan et al. engineered a biomimetic CuFeSe_2_-LOD@Lipo-CM nanoagents composed of CuFeSe_2_ ultra-small nanocrystals, lactate oxidase (LOD), and GBM cell membrane protein containing liposome (Lipo-CM) by in situ methodology for the CDT of orthotopic GBM. The GBM cell membrane protein embedding allowed the nanoagents to cross the partially raptured BBB and accurately target GBM because of their exceptional targeting and immune escaping capabilities. The LOD led to the elevation of in situ H_2_O_2_ contents for CDT by the oxidation of lactic acid to H_2_O_2_ and pyruvate present in the tumor. Meanwhile, the H_2_O_2_ is converted into toxic ^•^OH by the Fenton reaction of Cu-based nanoagents. Moreover, they applied NIR-II laser energy based on the photothermal efficiency of the nanoagents to induce in situ hyperthermia and enhanced CDT of the orthotopic GBM with minimum toxicity (Figure 5) [52].

### 5.3. Mn-Based Nanomaterials for CDT of GBM

Manganese oxide (MnO_x_), the Mn-based Fenton nanomaterials are activated to produce oxygen, alleviating hypoxia in the TME. This distinguishes it from Fe- and Cu-based Fenton reagents, as well as other Mn-based Fenton reagents. Furthermore, by decreasing intracellular GSH, MnO_x_, a traditional Fenton-like reagent, can increase CDT efficacy. Meanwhile, Mn^2+^ has shown promise as a tumor T_1_-weighted MRI contrast agent. Additionally, it possesses excellent photoacoustic imaging (PAI) and ultrasonic imaging capabilities [72].

**Figure 6:**
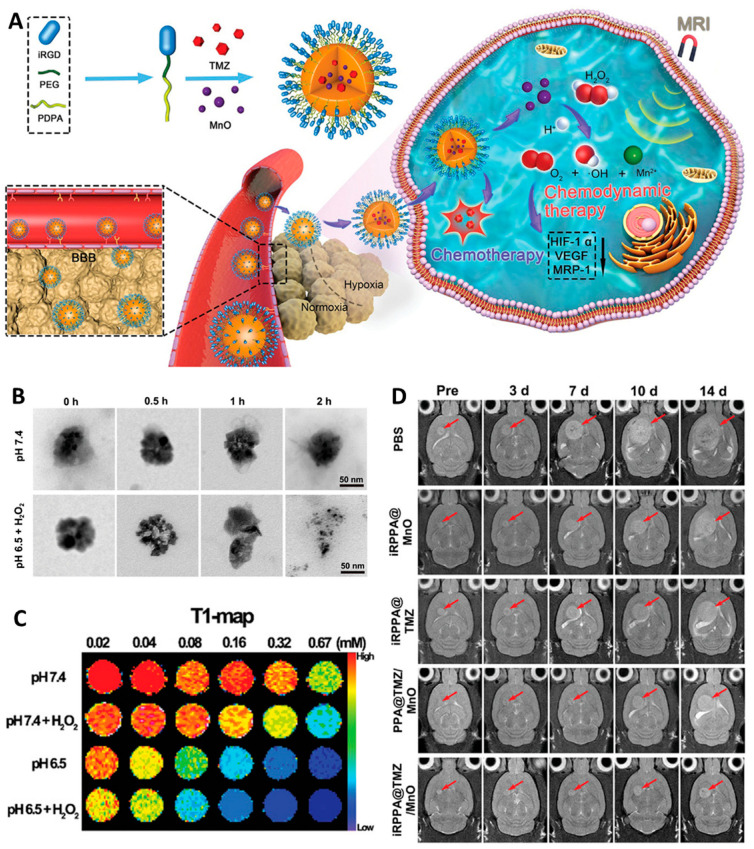
(**A**) Diagrammatic representation of Mn-based for image-guided multimodal chemotherapy/CDT for the treatment of GBM. (**B**) Transmission electron microscopy images demonstrating the structure of Mn-based nanomaterials at different pH levels. (**C**) In vitro MRI T_1_ map of Mn-based nanomaterials at different concentrations. (**D**) T_2_-weighted MRI figures of GBM within two weeks after treatment with different Mn-based nanomaterials [47]. Reproduced with permission from [47] Copyrights 2020, WILEY.

A recent study by Xiao et al. described the use of MnO_2_ nanomaterials and cisplatin co-loaded macrophages membrane-coated polymer nanogels (MPM@P NGs) to focus on chemotherapeutic and CDT [49]. Redox-responsive nanogels with disulfide bond cross-linkers were used to release cisplatin and deplete GSH, enhancing CDT in the tumor microenvironment. Additionally, MnO_2_ concurrently ingested the very rich natural GSH to facilitate the synthesis of ^•^OH, and the resulting Mn^2+^ catalyzed the breakdown of H_2_O_2_ to produce ROS for tumor apoptosis. T_1_-MRI was also utilized in this process. Additionally, the surface-expressed integrins α4, β1, and macrophage-1 antigen for effective glioma localization allow the polymer nanogels to pass through the BBB because of the outer macrophage membrane. Thus, these nanoplatforms offered a workable means of augmenting the synergistic antitumor effect guided by MRI. Wang et al. employed porous poly (lactic-co-glycolic acid) PLGA nanoparticles as templates to synthesize hollow manganese dioxide (HMnO_2_) shells in situ. These shells were then used to deliver bufalin and covered with a platelet membrane to obtain more precise cancer detection and successful therapy [73]. The P-selectin and CD44 receptor interaction inhibits angiogenesis and cancer cell growth, and enhances tumor localization via bufalin and platelet modification. Furthermore, HMnO_2_ nanoparticles were quickly broken down to enable the controlled release of bufalin in the presence of an acidic pH of the tumor and a comparatively high GSH concentration, while Mn^2+^ was acquired for further tailored chemotherapy, CDT, and MRI. These developments show that the rational design of inorganic nanomaterials for effective anticancer treatment can alter therapeutic platforms based on Fenton reactions [74]. In another study, the scientist engineered monodispersed nanoparticles of oleic-based manganese monoxide using a modified solvothermal technique. These nanoparticles were then enclosed in polymeric micelles with temozolomide (TMZ) and modified with iRGD peptide. The iRGD peptide-containing nanoparticles can enter tumor blood vessels and tissue by interacting with αvβ3 integrin and NRP-1 and can cross the BBB to target glioma cells. These versatile nanoparticles respond to the TME of glioma, releasing TMZ, Mn^2+^, and O_2_ simultaneously. The released TMZ induces apoptosis in tumor cells, while Mn^2+^ induces intracellular oxidative stress leading to tumor cell death. The released O_2_ alleviates tumor hypoxia and enhances the chemotherapy/chemodynamic therapeutic effect against glioma. Additionally, Mn^2+^ can be used as an MRI contrast agent to monitor tumors during treatment (Figure 6) [75].

## 6. Multimodal Therapy

In addition to the conventional GBM treatment modalities, newer therapeutic approaches such as photodynamic therapy (PDT), CDT, sonodynamic therapy (SDT), and photothermal therapy (PTT) have been met recently with much interest because of their negligible adverse outcomes and good selectivity [76,77]. However, the low H_2_O_2_ in TME and the lower acidity inside the cell make it impossible for the exceptionally effective Fenton response to occur, which results in an inadequate treatment effect from CDT. Consequently, the development of a Fenton agent that can both modulate intracellular acidity and deliver H_2_O_2_ on its own is very desirable for highly effective CDT. To address these concerns, scientists have designed smart materials to have exceptional multimodal GBM treatment capability. Cu-based nanomaterials exhibit a larger reaction rate and stronger production of ^•^OH when compared to standard Fe-based Fenton medicines. They also work better in TMEs that are somewhat acidic. So, the Cu-based smart materials can be applied for multimodal GBM treatment [78].

**Figure 7:**
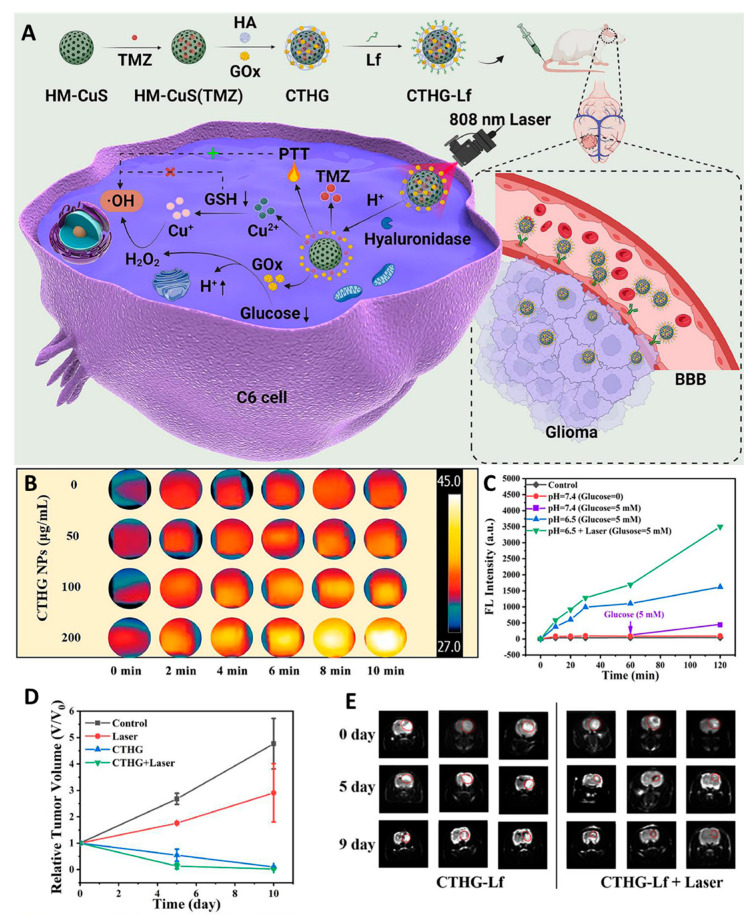
(**A**) Diagrammatic representation of the therapeutic process of applying smart nanomaterials for multimodal GBM therapeutic modality. (**B**) The in vitro photothermal therapy setup sample model and the IR thermal images of smart nanomaterials at different intervals. (**C**) ^•^OH production in various conditions. (**D**) Demonstration of the tumor volume after treatment for 9 days. (**E**) In vivo MRI of the mice bearing tumor [83]. Reused under Creative Commons Attribution License.

Hollow mesoporous copper sulfide (HM-CuS) nanoparticles have garnered greater interest in recent years because of their exceptional adaptability. For instance, due to its hollow shape, it can be used to provide highly selective PTT as well as act as a drug carrier [79,80]. It has been demonstrated that building a medication route of administration that responds to signals is a viable method for obtaining controlled medication release. The internal lysosomal enzyme hyaluronidase (Hyal) can degrade the extracellular matrix ingredient hyaluronic acid (HA) [81]. As a result, it is a strong contender for the role of gatekeeper because it can both reach the tumor location with Hyal-1, which is amenable to drug release on need and stops the medication from leaking too soon. When exposed to an 808 nm laser light and an acidic TME, HM-CuS nanoparticles used a redox process to split the additional Cu^2+^ and deplete GSH. The Cu^2+^ produced is then used to convert H_2_O_2_ into extremely toxic ^•^OH along with a more effective Fenton response for CDT. For starvation therapy (ST), GOx can compete with tumor cells for glucose. It can also facilitate the oxidation process reaction of glucose to produce gluconic acid and H_2_O_2_ effectively, enhancing the amount of H_2_O_2_ and acidic TME at the same time to speed up the Fenton reaction. Consequently, the combination of multifunctional HM-CuS nanoparticles, enzyme-responsive HA, and GOx would result in extremely effective multimodal synergistic treatment and on-demand drug release. Motivated by the above-mentioned concern, scientists created the intelligent BBB-permeable nanoplatforms (CTHG-Lf) in this study, utilizing HA as the gatekeeper and HM-CuS nanoparticles as TMZ carriers. Additionally, they modified the platform further by adding GOx and lactoferrin (Lf) to achieve very effective complementary chemotherapy (CT), ST, CDT, and PTT of GBM. With the use of acidic TME and 808 nm laser irradiation, HM-CuS nanoparticles, a Fenton-like substance, exhibited good CDT action. Lf, a cationic Fe-binding glycoprotein found in mammals, gives CTHG-Lf nanoparticles the capacity to target GBM cells and effectively penetrate the BBB through RMT [82]. In addition to preventing premature drug leakage, modification of the HA surface on HM-CuS nanoparticles results in an important message from TMZ at the tumor location. H_2_O_2_ and gluconic acid can be obtained through GOx-mediated ST, which enhances the therapeutic impact of CDT. Furthermore, when CTHG-Lf nanoparticles are exposed to an 808 nm NIR light, they exhibit a modest photothermal effect that can induce PTT, speed up the Fenton reaction, and improve drug administration by increasing blood flow. This allows for the synergistic use of CT, ST, CDT, and PTT to inhibit the development of GBM (Figure 7).

He et al. designed a lipopolysaccharide-free bacterial membrane camouflaged biomimetic smart nanomaterial for multimodal therapy of orthotopic GBM. Because of the immune-escaping ability and BBB crossing capacity of the bacterial outer membrane, the smart biomimetic nanomaterial targeted the cancer cells and accumulated in the tumor site. The GOx rapidly consumed the glucose present in the tumor cells to produce the H_2_O_2_ and gluconic acid for ST. The excessive amount of H_2_O_2_ produced by the GOx enzyme-catalyzed reaction continuously supplied the Cu_9_S_8_-mediated Fenton reaction to enhance CDT. Meanwhile, GOx-induced depletion of oxygen created the hypoxic condition TME and activated AQ4N prodrug for CT. The increased temperature in the local tumor area due to the absorption of NIR-II radiation by Cu_9_S_8_ upon NIR-II laser irradiation helped to achieve photothermal-enhanced CDT, increased GOx activity, and accelerated drug release. The results demonstrated that the biomimetic smart nanomaterials were promising multifunctional nanoagents for efficient multimodal treatment of GBM [84].

## 7. Challenges in Chemodyanamic Therapy of Brain Tumor

Even though CDT has been demonstrated as an advanced therapeutic strategy for treating GBM due to its exceptional biocompatibility, effective tumor targeting, long-term therapeutic efficacy, and absence of activation stimuli, the intrinsic barriers of the TME and BBB are still preventing CDT from being developed further and used in clinical settings. Fortunately, new nanosystems that have the potential to overcome those CDT problems have been built and investigated, owing to the tremendous growth of nanoscience and nanobiotechnology. However, before more clinical applications, a few issues, which are discussed in the following sections, still need to be resolved.

### 7.1. Biosafety Concerns

At the cellular level, there are numerous ways in which CDT-based nanosystems might penetrate cells, potentially causing modifications or even the complete cessation of normal cellular operations. This could result in needless harmful side effects and problems with biocompatibility [85]. Particularly, the majority of the inorganic or hybrid nanomaterials used in the already documented CDT-based nanosystems have the potential to trigger an in vivo immunological response. As a result, there are serious concerns about the biosafety of CDT-based nanosystems in clinical applications. Keeping in mind the above-mentioned problems, there is an utmost need to design biocompatible materials that have minimum side effects on the natural physiological phenomenon [86].

Smart biomolecules having maximum biocompatibility should be used in designing the nanosystems for effective CDT of GBM. For example, biomimetic systems, bacterial cell membrane-coated Fenton nanosystems, cancer cell membrane-coated nanoagents, and protein-coated nanomaterials are the representative smart Fenton nanosystems for biocompatible CDT.

### 7.2. Mechanism

Although numerous studies have been conducted on the Fenton contributing processes that generated ROS, the catalysis procedure, and the resulting structural destruction caused by ROS remain poorly understood, which makes it challenging to logically enhance and maximize catalytic efficiency. To inform cancer treatment, a thorough understanding of the in vivo CDT process and its associated processes is essential [87]. To better understand the true value of CDT for tumor therapy, for instance, methods that can track the Fenton reaction in vivo are required. Moreover, the identification of ROS generated during the Fenton reaction is also another mystery to explore. The mechanism to cross the molecular pathways, BBB, and membrane channels should be explained comprehensively [88]. The understanding of the mechanism of the Fenton reaction and CDT will provide us with the insight and knowledge to modulate the structure and morphology of the nanomaterials for enhanced CDT.

### 7.3. Complexity of Nanosystems-Based CDT

The current research shows that although CDT-based nanosystems are quite complex in design, they are rarely employed in clinical settings. On the one hand, because the chemical compositions of complex nanosystems are too complex to accurately forecast their biocompatibility, they are typically linked to biological toxicity [89]. Conversely, the synthesis of CDT-based nanosystems has only been documented at the laboratory scale; however, for real-world uses, the repeatability is insufficient for large-scale industrial implementation. Thus, there has been a lot of interest in developing CDT-based nanosystems with straightforward structures, stable compositions, and effective responsiveness to endogenous and/or external stimuli [90]. In this modern era of advanced technology, it is a challenge for researchers to design simple and smart materials that have maximum biocompatibility and therapeutic ability. The simple structure will provide insight and understanding of the therapeutic mechanism, and it is also easy to control and modulate the chemistry of the smart material used.

### 7.4. Delivery

Fenton metal can be loaded using nanoscale delivery methods for tailored delivery through the EPR effect. Typically, this passive targeting is insufficient for a CDT-like complex therapeutic modality. By adding more ligands to the particle surface that are specifically designed to recognize tumors, active targeting can be achieved [91]. Additionally, computational systems utilizing CDT include an excellent way to enhance the results of tumor therapy; however, integrating CDT with several therapies into a single set usually necessitates laborious preparation steps and specialized material design. Developing multimodal nanoplatforms that are resilient, cost-effective, and simple is still considered crucial to achieving synergistic benefits in combinatorial medicine [92]. Moreover, the Fenton nanomaterials coated with bacterial cell membranes, lipoproteins, cancer cell membranes, and lipopolysaccharides can selectively be targeted to the cancer area.

### 7.5. pH of TME

The required pH range for an effective Fenton reaction is 2–4, while the pH of TME usually vary between 6.85 and 5. This scenario demonstrated that Fenton reaction cannot use the full potential of TME and inhibit the efficacy of CDT. To explore the significant efficiency of Fenton reaction by utilizing TME, scientists should consider pH of the tumor area carefully. For example, the pH of vaginal tissues is about 4, which means that Fenton reaction can be used for the treatment of cervical cancer. Moreover, the pH of the stomach tissues is about 2 and Fenton reaction can also be applied for the treatment of gastric cancer.

## 8. Conclusions

The intriguing CDT approach uses the Fenton process, in which endogenous H_2_O_2_ reacts with Fenton metal ions that come out of metal–organic complexes (Cu^+^ and Mn^2+^) to produce highly cytotoxic ^•^OH at tumor locations, which has an anti-cancer impact. When combined, CDT offers an extremely attractive strategy for theranostic treatments related to brain tumors, which have attracted a lot of research attention to accelerate the advancement in the therapeutic realm. It has been determined that CDT is a form of ROS therapy that works by operating Fenton reactions using low pH and overexpressed H_2_O_2_ of TME, and then produces cytotoxic ^•^OH to cause oxidative damage to tumor cell protein, DNA, phospholipids, mitochondria, etc. A major factor in the development of CDT is the variety of methods in designing the materials for Fenton reactions. Due to the complexity of TME, traditional Fenton reaction-based chemodynamic medicines produce insufficient amounts of harmful ROS to completely eradicate tumors. Modifying the chemodynamic drug design for enhanced Fenton or Fenton-like response can result in decreased adverse effects and higher therapeutic efficacy. CDT is regarded as a harmless therapeutic approach with minimal adverse reactions and superior tumor susceptibility when compared to conventional tumor treatment. More nanosystems that can overcome the difficulties of CDT have been devised and explored as a result of the quick growth of nanoscience and nanobiotechnology. However, as previously stated, significant work must be conducted at the fundamental and animal/clinical levels to have a clinical impact, which could spur further advancements in the field. In general, research in this field remains in its early stages, and more research has to be conducted before it can be potentially applied in practice. Future studies should concentrate on practical applications of CDT and CDT-based therapeutic approaches. Although there is still a long way to go until CDT is clinically implemented, we anticipate that more tumor patients will benefit from this treatment and that new nanosystems will be built for it.

## Figures and Tables

**Table 1:** Recent Summary of Nanomedicines Applied for the CDT of GBM.

Year	Nanomedicine	Treatment Method	ROS Type	Reference
2020	iRPPA@TMZ/MnO	CDT/CT	^•^OH	Tan et al. [47]
2021	GOx@MnCaP@fibrin	ST/CDT	^•^OH	Li et al. [48]
2021	MPM@P NGs	CDT/CT	^•^OH	Xiao et al. [49]
2022	TA-VOx NBs	PTT/CDT	^•^OH	Guo et al. [50]
2022	MPC@siBcl-2	CDT	^•^OH	Zhang et al. [51]
2022	CuFeSe_2_-LOD@Lipo-CM	CDT	^•^OH	Pan et al. [52]
2023	NaGdF_4_:Yb, Tm@NaYF_4_:Yb, Nd@NaYF_4_	CDT/PDT/CT	^•^OH/^•^O_2_^−^	Lv et al. [53]
2023	AG@Cu9S8@dOMV	CDT/PTT/CT	^•^OH	He et al. [54]
2023	CS–P@CM NPs	CDT/ST	^•^OH	Wang et al. [55]
2024	FMDM@P NGs	CDT/CT	^•^OH	Huang et al. [56]
